# Treatment of Breast Cancer-Bearing BALB/c Mice with Magnetic Hyperthermia using Dendrimer Functionalized Iron-Oxide Nanoparticles

**DOI:** 10.3390/nano10112310

**Published:** 2020-11-22

**Authors:** Marzieh Salimi, Saeed Sarkar, Mansoureh Hashemi, Reza Saber

**Affiliations:** 1 School of Physics and Astronomy, University of Exeter, Exeter EX4 4QL, UK; 2 Department of Medical Physics and Biomedical Engineering, Tehran University of Medical Sciences, Tehran 1417613151, Iran; sarkar@tums.ac.ir; 3 Research Center of Science and Technology in Medicine, Tehran University of Medical Sciences, Tehran 14185-615, Iran; 4 Functional Neurosurgery Research Center, Shahid Beheshti University of Medical Sciences, Tehran 1989934148, Iran; mansoureh.hashemi@sbmu.ac.ir; 5 Department of Medical Nanotechnology, School of Advanced Technologies in Medicine, Tehran University of Medical Sciences, Tehran 1417755469, Iran; rsaber@sina.tums.ac.ir

**Keywords:** dendrimer, iron oxide nanoparticles, magnetic hyperthermia, breast cancer

## Abstract

The development of novel nanoparticles for diagnostic and therapeutic applications has been one of the most crucial challenges in cancer theranostics for the last decades. Herein, we functionalized iron oxide nanoparticles (IONPs) with the fourth generation (G_4_) of poly amidoamine (PAMAM) dendrimers (G_4_@IONPs) for magnetic hyperthermia treatment of breast cancer in Bagg albino strain C (BALB/c)mice. The survival of breast cancer cells significantly decreased after incubation with G_4_@IONPs and exposure to an alternating magnetic field (AMF) due to apoptosis and elevation of Bax (Bcl-2 associated X)/Bcl-2(B-cell lymphoma 2) ratio. After intratumoral injection of G_4_@IONPs, tumor-bearing BALB/c mice were exposed to AMF for 20 min; this procedure was repeated three times every other day. After the last treatment, tumor size was measured every three days. Histopathological and Immunohistochemical studies were performed on the liver, lung, and tumor tissues in treated and control mice. The results did not show any metastatic cells in the liver and lung tissues in the treatment group, while the control mice tissues contained metastatic breast cancer cells. Furthermore, the findings of the present study showed that magnetic hyperthermia treatment inhibited tumor growth by increasing cancer cell apoptosis, as well as reducing the tumor angiogenesis.

## 1. Introduction

Breast cancer is the most common cancer in women both in developed and developing countries. In 2018, over 626,679 women died from breast cancer [1,2]. The most common treatment methods for breast cancer, e.g., radiation therapy, surgery, and chemotherapy, possess several side effects, such as secondary cancer, tumor recurrence, and normal tissue damage. Magnetic hyperthermia as a nanotechnology-based method for cancer treatment has attracted lots of attention in recent years [3,4,5,6,7].

In magnetic hyperthermia treatment, magnetic nanoparticles (MNPs) are injected into the tumor and subsequently exposed to an alternating magnetic field (AMF) to transform the magnetic field to heat through several physical mechanisms. Consequently, the temperature of the tumor tissue rises 5–7 °C above the normal temperature of the body [8]. In this situation and also considering that cancer cells are more sensitive to heat, these cells will be damaged while healthy cells can be spared [9,10].

The outcome of magnetic hyperthermia treatment depends on MNPs characteristics, such as saturation magnetization, polydispersity, and aggregation [11]. In this regard, iron oxide magnetic nanoparticles (IONPs) are suitable candidates for magnetic hyperthermia due to their high saturation magnetization. On the other hand, one of the major challenges in using IONPs in biomedical studies, in particular magnetic hyperthermia, is their tendency to agglomerate due to the high surface area to volume ratio [12,13]. To overcome this issue, it is necessary to use a polymeric coating that first prevents IONPs aggregation, and secondly possesses multiple surface-active functional groups for conjugation to the targeting, imaging, and therapeutic molecules.

Dendrimers are highly branched, star-shaped macromolecules with nanometer-scale architecture, and well suited for biological applications, such as gene delivery, drug delivery, photodynamic therapy, and imaging [4,13,14,15,16,17]. Dendrimers are composed of three components: a central core, an internal dendritic structure called generation, and an external surface with functional groups [18]. Monodisperse dendrimers are synthesized by step-wise chemical approaches (divergent and convergent) to give distinct generations of molecules with uniform size and shape, and multiple surface groups [19]. Poly amidoamine (PAMAM) dendrimers possess a condensed outer amine shell which can react with biomolecules and prevents the agglomeration of IONPs [20]. We have reported the synthesis method of functionalized IONPs with fourth-generation PAMAM dendrimers (G_4_@IONPs) in our previous studies [21]. The results showed that the dendrimer coating prevented IONPs aggregation and increase their colloidal stability. 

Many studies were performed on magnetic hyperthermia using different functionalized MNPs on animal models of cancer [22,23,24,25,26,27,28,29,30,31,32,33,34,35] (Table 1). Generally speaking, their results were promising and showed a significant remission in the tumor size after treatment. Furthermore, several histological damages and destructions were detected in the treated tumor tissues. Regarding dendrimers are the powerful tools in theranostic applications and a lack of studies using dendrimer functionalized MNPs for magnetic hyperthermia, this study was performed to investigate the therapeutic effects of magnetic hyperthermia using G_4_@IONPs on breast cancer tumor.

**Figure 1:**
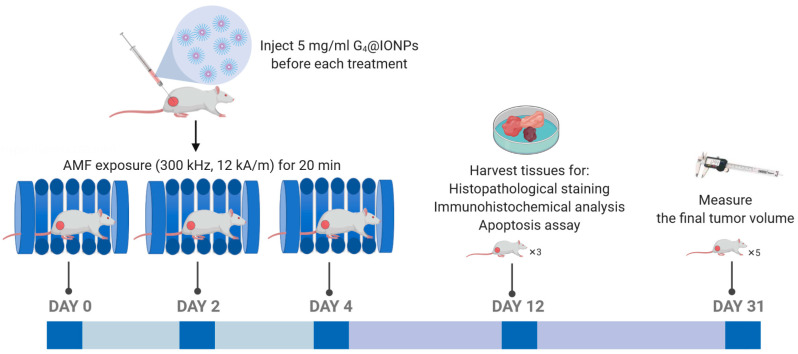
Experimental workflow for magnetic hyperthermia treatment in breast cancer-bearing BALB/c mice.

In the present study, G_4_@IONPs were synthesized via a co-precipitated method and functionalized by adding methyl acrylate/ethylenediamine stepwise to the G_0_@IONPs solution. Subsequently, the polyethylene glycol coating (PEGylation) procedure was performed to increase MNPs stability. G_4_@IONPs were characterized by transmission electron microscope (TEM), Fourier-transform infrared spectroscopy (FTIR), and zeta potential measurements. The cytotoxicity of G_4_@IONPs in mice estrogen receptor-positive breast carcinoma cells (MC_4_L_2_) was evaluated by 3-[4, 5-dimethylthiazol-2yl]-2, 5 diphenyl tetrazolium bromide (MTT) assay. For magnetic hyperthermia treatment, MC_4_L_2_ were cultured with G_4_@IONPs for 2 h and then exposed to the AMF for 120 min. MTT assay, terminal deoxynucleotidyl transferase-mediated dUTP nick-end labeling (TUNEL) staining, and real-time polymerase chain reaction (PCR) were performed to investigate the efficiency of treatment. Breast cancer-bearing BALB/c mice were exposed to the AMF for 20 min after intratumoral G_4_@IONPs injection. The tumor volume and mortality of mice in all groups were monitored after the last treatment for four and eight weeks, respectively. Liver, lung, and tumor tissues were harvested from control and treated mice eight days after the last treatment and assessed by histopathological staining. Furthermore, apoptosis and angiogenesis in control and treated tumor tissues were studied by TUNEL and Immunohistochemistry assays, respectively (Figure 1). The results showed that magnetic hyperthermia using G_4_@IONPs could effectively demolish breast cancer cells by increasing apoptosis and inhibit tumor growth by reducing the tumor angiogenesis and perfusion.

## 2. Materials and Methods 

### 2.1. Chemicals and Materials

*F*erric chloride hexahydrate (FeCl_3_, 6H_2_O, 99% *w/w*), ferric sulfate heptahydrate (FeSO_4_·7H_2_O, 99% *w/w*), hydrochloric acid (HCl, 32% *v/v*), methanol (99.9% *v/v*), ammonia solution (NH_3_, 32% *v/v*), 3-aminopropyltriethoxysilane (NH_2_(CH_2_)_3_-Si-(OCH_3_)_3_, methyl acrylate (99.5% *v/v*), (3-Aminopropyl)triethoxysilane (APTS), ethanol (99.9% *v/v*), methoxy-PEG and ethylenediamine (99% *v/v*), fetal bovine serum (FBS), dimethyl sulfoxide (DMSO), Eagle’s minimal essential medium (DEMEM), and PenStrep were purchased from Sigma-Aldrich (Hamburg, Germany).

### 2.2. Magnetic Nanoparticles Synthesis

**Figure 2:**
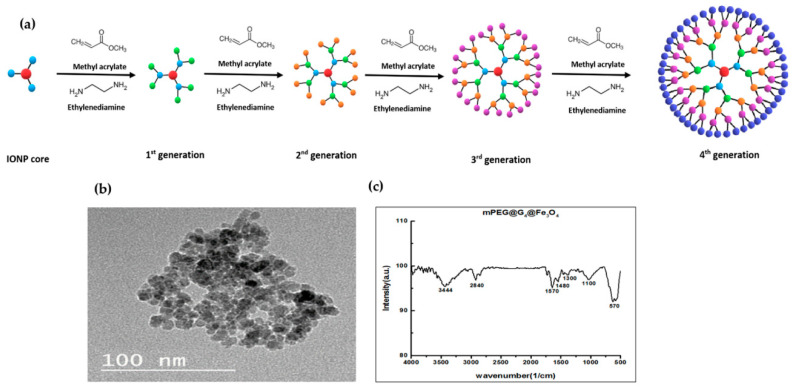
(**a**) Schematic illustration of functionalization of iron oxide magnetic nanoparticles (IONPs) with G4 poly amidoamine (PAMAM) dendrimers, (**b**) transmission electron microscope (TEM) image of G_4_@IONPs, (**c**) Fourier-transform infrared spectroscopy (FTIR) spectra for PEGylated G_4_@IONPs.

G_4_@IONPs synthesis and characterization were explained in detail in our previous studies [21,36]. Briefly, IONPs were synthesized by co-precipitation of 0.84 g of FeSO_4_ and 1.22 g of FeCl_3_ and then functionalized by PAMAM dendrimers with step by step addition of methyl acrylate and Ethylenediamine (Figure 2a). 50 mL methyl acrylate/methanol solution (20%, *v/v*) was added to the 10 mL ethanol solution of APTS coated IONPs (5 g/L); after 1h sonication and stirring, 15 mL ethylenediamine/methanol (50%, *v/v*) was added to the previous solution followed by 3 h sonication at room temperature. Subsequently, methoxypolyethylene glycol (mPEG) molecules (molecular weight = 4000 Da) having three times the mass of the iron were dissolved in ethanol and added to the G_4_@IONPs solution before 18 h reflux. Transmission electron microscopy (TEM) and Fourier transform infrared (FTIR) spectroscopy were applied to assess G_4_@IONPs size and presence of PAMAM bonds on the IONPs surface, respectively. In addition,, the surface charge of G_4_@IONPs was measured using a Zetasizer instrument (Malvern Panalytical, Malvern, UK).

### 2.3. Cell Culture

MC_4_L_2_ breast cancer cells were obtained from the Pasteur Institute (Tehran, Iran) and cultured in DMEM medium supplemented with 10% (*v/v*) FBS, and 1% Pen-Strep at 37 °C and 5% CO_2_. Cytotoxicity of G_4_@IONPs Cytotoxicity of G_4_@IONPs was evaluated by MTT assay. After 24 h incubation, MC_4_L_2_ cells were washed with PBS twice and treated with different G_4_@IONPs concentrations of 1500, 1000, 500, 100, 10, and 0/control µg/mL for 24 h. The culture media were then removed, and the MTT solution was added to each well for 4 h. Finally, 100 µL DMSO was added and the absorbance of wells was read using an ELISA (enzyme-linked immunosorbent assay) plate reader (Hyperion, microplate reader MPR4+) at 540 nm [37].

### 2.4. Magnetic Hyperthermia Treatment in Cancer Cells

**Figure 3:**
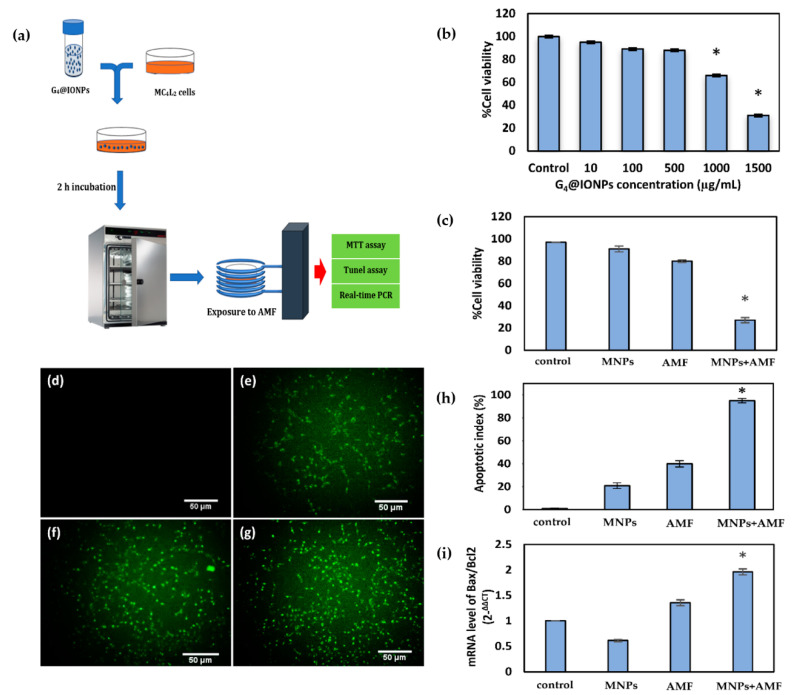
(**a**) Schematic illustration of magnetic hyperthermia in vitro experiments. 2 × 105 MC_4_L_2_ cells were seeded in a 35 mm culture dish overnight. 500 µg/mL G_4_@IONPs was added to cell culture media. After 2h incubation, cells were exposed to alternating magnetic field (AMF) for 120 min. MTT and TUNEL assays, and real-time polymerase chain reaction (PCR) were performed to assess cell viability, apoptosis and Bax/Bcl2 ratio immediately after treatment; (**b**) Cytotoxicity of G_4_@IONPs in MC_4_L_2_ cells (*P < 0.05); (**c**) MC_4_L_2_ cells viability percentage after magnetic hyperthermia treatment (*P < 0.05); TUNEL staining showed the apoptotic cancer cells in groups of: (**d**) control; (**e**) magnetic nanoparticles (MNPs); (**f**) AMF; (**g**) MNPs + AMF; (**h**) Apoptotic index in all groups (*P < 0.05); (**i**) Bax/Bcl-2 ratio in MC_4_L_2_ cells after magnetic hyperthermia treatment (*P < 0.05).

MC_4_L_2_ cells were divided into four experimental groups: MNPs + AMF, AMF, MNPs, and control. In the group of MNPs + AMF, cells were cultured with 500 μg/mL G_4_@IONPs for 2 h and then exposed to the AMF (300 kHz and 12 kA/m; LABA, HT-1000W, Nanotechnology System Corporation (NATSYCO), Tehran, Iran) for 120 min. The other cells were only exposed to the AMF (AMF group) or cultured with 500 μg/mL G_4_@IONPs (MNPs group). Control cells received neither G_4_@IONPs nor AMF exposure. MTT assay was performed to assess the cellular viability in all group (Figure 3a). 

### 2.5. G_4_@IONPs Cellular Uptake and Localization

Cellular uptake of G_4_@IONPs was evaluated by Prussian blue staining and measured by Inductively Coupled Plasma Mass Spectrometry (ICP-MS) [38,39]. Different concentrations of G_4_@IONPs (500, 250, 100, 50, and 0 (control) μg/mL) were added to cell culture media. After 2 h incubation, cells were fixed with 4% formalin and incubated with 4% potassium ferrocyanide and 4% hydrochloric acid (50%, *v/v*) for 20 min. Finally, the G_4_@IONPs cellular localization was observed by optical microscopy (Olympus, Tokyo, Japan). Furthermore, MC_4_L_2_ cells were trypsinized after 2 h incubation with G_4_@IONPs (500, 250, 100, 50, and 0 (control) μg/mL) and lysed by 2 mL 65% nitric acid; the quantity of cellular uptake of G_4_@IONPs was assessed using ICP-MS (Varian Inc, Palo Alto, CA, USA). To obtain the iron concentration per cell, the total iron concentration measured by ICP-MS was divided by the number of lysed cells.

### 2.6. Apoptotic Cell Death Assessment

Apoptosis in cancer cells after magnetic hyperthermia treatment was assessed by terminal deoxynucleotidyl transferase dUTP (2’-deoxyuridine-5’-triphosphate) nick end labeling (TUNEL) assay. Briefly, MC_4_L_2_ cells were fixed by 4% paraformaldehyde for 10 min and permeabilized with 0.2% Triton X-100 for 2 min on the ice and then incubated with TUNEL reaction mixture. For the positive and negative control, cells were treated with 5% ethanol and label solution, respectively [40]. The apoptotic index was the number of apoptotic cells divided by the total number of cells.

### 2.7. Effect of Magnetic Hyperthermia Treatment on the Expression of Apoptosis-related Genes

Expression of Bax and Bcl-2 in MC_4_L_2_ cells was measured with real-time PCR followed by magnetic hyperthermia treatment. Complementary DNA (cDNA) was synthesized using RevertAid First Strand cDNA Synthesis Kit (Fermentas, Germany) based on the manufacturer’s protocol. Quantification of gene expression was done with RotorGene 6000 detection system (Corbett Research, Australia). PCR solution (20 μL) was composed of 2 μL cDNA, 4 μL of master mix solution of 5 × HOT FIREPol^®^ EvaGreen^®^ qPCR Mix Plus kit (ROX), and 0.5 μL of each primer. The Bax/Bcl-2 level was normalized to the glyceraldehyde 3-phosphate dehydrogenase (GAPDH, housekeeping gene) transcript and calculated utilizing the 2^−ΔΔCt^ method (Table 2) [41]. 

### 2.8. Ethical Statement and Animal Welfare

The animal experiments were approved by the Animal Ethics Committee of Tehran University of Medical Sciences (IR.TUMS. REC.28169); Applied Research Ethics National Association guidelines were administered for animal welfare. Six- to eight-week-old female BALB/c mice (25–30 g) were purchased from the Animal Center of Pasture Research Center. The groups of five were kept in the individual cages with unlimited access to water and food, and the circadian rhythm was 12 h in the light and 12 h in the dark. 

### 2.9. Breast Tumor Induction in BALB/c Mice

MC_4_L_2_ cells (1 × 10^6^/0.1 mL) were injected into the right inguinal flank of the female mice under ketamine and xylazine anesthesia. The tumor size was measured regularly using a digital Vernier caliper (Mitutoyo, Kawasaki, Japan) and calculated using the following equation:

*V* (mm^3^) = (*L* × *W^2^*) × 0.5,

where *V* = tumor volume, *L* = Length, and *W* = Width.

Two weeks post cell injection, the mice with the tumor ≥ 50 mm^3^ were taken and randomly divided into four groups: control, G_4_@IONPs injection (MNPs), AMF exposure (AMF), and treatment (MNPs + AMF).

### 2.10. Magnetic Hyperthermia Treatment in BALB/c Mice

G_4_@IONPs (5mg/mL) were injected intratumorally under ketamine and xylazine anesthesia; then, the mouse was transferred into the magnetic coil and exposed to the AMF (300 kHz and 12 kA/m) for 20 min. The treatment procedure was repeated three times for each animal of the MNPs+AMF group, every other day.

### 2.11. Histopathological Studies in Liver, Lung, and Tumor Tissues

Three mice in each group were euthanized eight days post-treatment. Harvested tissues (liver, lung, and tumor) were fixed in the 10% NBF (neutral buffered formalin, pH 7.26) for 48 h, then processed and embedded in paraffin. The 5 µm thick sections were prepared and stained with Hematoxylin and eosin (H&E). The histological slides were evaluated by an independent reviewer, using light microscopy (Olympus, Tokyo, Japan). Histopathological evaluation was performed using the Nottingham histologic grading system (Elston-Ellis modification of the Scarff-Bloom-Richardson grading system) for breast cancer [42]. This scoring system grades the breast tumor malignancy between 3 and 9 based on the following features: the amount of gland formation (acinar or tubular differentiation), the nuclear features (pleomorphism), and the mitotic activity, which were scaled from 1–3. A tumor with a sum of 3–5 was considered as Grade 1 (well-differentiated). A tumor with a sum of 6–7 and 8–9 was considered as Grade 2 (moderately differentiated) and Grade 3 (poorly differentiated), respectively. Moreover, any changes, including metastasis, inflammatory response, coagulative necrosis, hemorrhage, and hyperemia, were assessed in different groups, comparatively. 

### 2.12. Immunohistochemistry (IHC) Assay in Tumor Tissues

The angiogenesis index in tumor tissues was assessed using a monoclonal mouse anti-human cluster of differentiation 34 (CD_34_) antibody (ready to use, Biocare, Pacheco, CA, USA) in control and treatment mice. This index was defined by counting the positive staining for CD_34_ in five fields at 200× magnification, using computer software Image-Pro Plus®V.6 (Media Cybernetics, Inc., Silver Spring, MD, USA), and the results were expressed as the mean number of vessels ± standard error of the mean (SEM). The negative control sections were obtained by omitting the primary antibody for CD_34_.

### 2.13. Apoptosis in the Tumor Tissues (TUNEL Assay)

TUNEL assay (TUNEL Assay Kit-BrdU-Red, ab66110) was utilized to determine if treatment inhibited the tumor tissue growth. The number of apoptotic cells was counted in three high-power fields (40× magnification), and the mean percentage of apoptotic cells was reported. 

### 2.14. Statistical Analysis

Results were obtained from three independent experiments and reported as the mean ± SEM. One-way analysis of variance (ANOVA) was used to compare the means in the groups. Statistical differences were significant when P < 0.05.

## 3. Results

### 3.1. Characterization of G_4_@IONPs 

G_4_@IONPs characterization has been explained in detail in our previous paper [21]. Briefly, the results of TEM showed that the size of nanoparticles was 10 ± 4 nm (Figure 2b). The surface charge of G_4_@IONPs determined by zeta potential measurement was +35 mV at pH = 7 and 25 °C. Zeta potential is an indicator of surface charge that can be used to predict the MNPs solution stability and also is a crucial parameter for the interaction of G_4_@IONPs with biological systems in vivo. FTIR demonstrated the presence of Fe_3_O_4_ core, dendrimer coating, and mPEG molecules in the G_4_@IONPs structure. Magnetite core was detected by a strong peak at 570 cm^−1^ [43]. The peaks at 1450, 1490, 1570, and 1620 cm^−1^ confirmed the existence of –CO–NH– bonds related to PAMAM dendrimer on the surface of IONPs. In addition,, the absorption bond at 2888 cm^−1^ and 1110 cm^−1^ was attributed to the C–H and C–O bond of mPEG, respectively (Figure 2c) [44,45,46]. 

### 3.2. Cytotoxicity of G_4_@IONPs in Cancer Cells (MTT Assay)

The MTT assay was performed to investigate the toxic effect of G_4_@IONPs on MC_4_L_2_ cells at different concentrations. The results showed that G_4_@IONPs did not have significant cytotoxicity at concentrations up to 500 µg/mL. The cell viability reduced significantly at 1000 and 1500 µg/mL (66% and 31%, respectively) (Figure 3b). 

### 3.3. Effect of Magnetic Hyperthermia Treatment on the Viability of Cancer Cells 

MTT assay results showed that magnetic hyperthermia treatment (HT + MNPs) significantly decreased cancer cells viability (41.7 ± 2.3%). Furthermore, cell viability was 91.3 ± 1.1% and 97.8 ± 2.6% in groups of MNPs-HT and HT-MNPs, respectively (Figure 3c). 

### 3.4. Cellular Apoptosis and Expression of Apoptosis-Related Genes after Magnetic Hyperthermia Treatment

The results of the TUNEL assay indicated that the number of apoptotic cells in the treatment group (MNPs + AMF) was significantly higher than that in the control group (apoptotic index = 86%). On the other hand, the number of apoptotic cells in groups of MNPs and AMF did not have a significant difference with the control group (14% and 27%, respectively) (Figure 3d–h). The expression of Bax in cancer cells increased significantly (*P* < 0.05) after magnetic hyperthermia treatment (MNPs + AMF) compared to that in control group; Furthermore, Bcl-2 expression decreased significantly (*P* < 0.05) in the group of MNPs + AMF (Figure 3i).

### 3.5. Cellular Uptake and Localization of G_4_@IONPs

**Figure 4:**
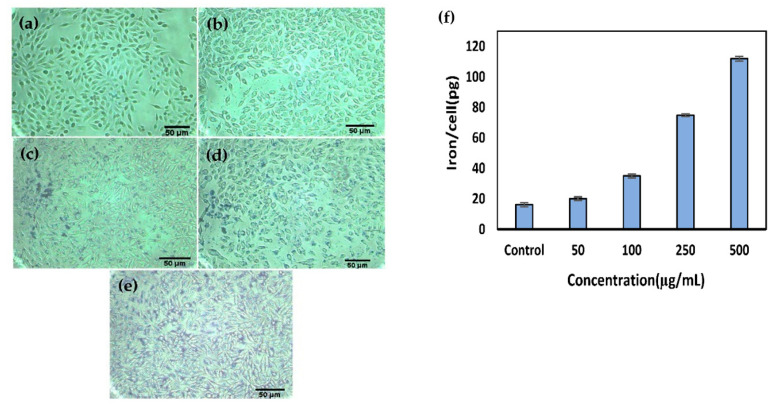
Prussian blue staining illustrated the density of iron oxide nanoparticles inside the MC_4_L_2_ cells after 2 h incubation with (**a**) control; (**b**) 50; (**c**) 100; (**d**) 250; (**e**) 500 µg/mL of G_4_@IONPs; the iron particles appeared as blue precipitates in the cell cytoplasm; (**f**) the cellular uptake of G_4_@IONPs at different concentrations was measured by Inductively Coupled Plasma Mass Spectrometry (ICP-MS).

Prussian blue staining was performed to demonstrate the cellular uptake of G_4_@IONPs after 2 h. The iron particles were seen as blue precipitates which increased in the cell cytoplasm with increasing the G_4_@IONPs concentration (Figure 4a–e). Consequently, ICP-MS results revealed that the iron concentration in the cells increased in a concentration-dependent manner. The amount of 16.1 ± 2.7 pg iron was detected in control cells (Figure 4f).

### 3.6. Histopathological Effects of Magnetic Hyperthermia Treatment on Liver and Lung Tissues

**Figure 5:**
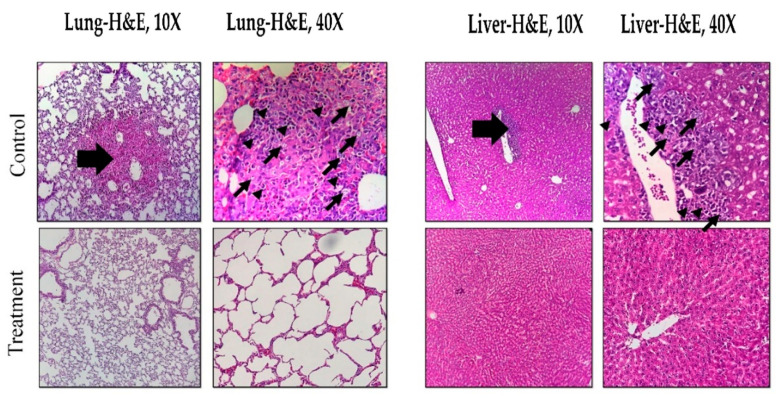
Histopathological images of lung and liver tissues in control and treatment (MNPs + AMF) groups. Thick arrows show microscopic tumor metastasis in lung and liver, thin arrows represent tumor cells, and arrowheads: inflammatory cells.

All H&E-stained lung and liver sections from control and treatment groups were evaluated histologically (Figure 5). Focal metastasis of breast cancer (stars) was seen in the control group (thick arrows). Moreover, hemorrhage, necrosis, and infiltration of inflammatory cells were detected in control tissues due to the invasion of tumor cells. The treatment group showed only mild edema in liver tissue. The histology of the lung tissue in the treatment group was normal without any significant histopathological change.

### 3.7. Histopathological Effects of Magnetic Hyperthermia Treatment on Tumor Tissue

**Figure 6:**
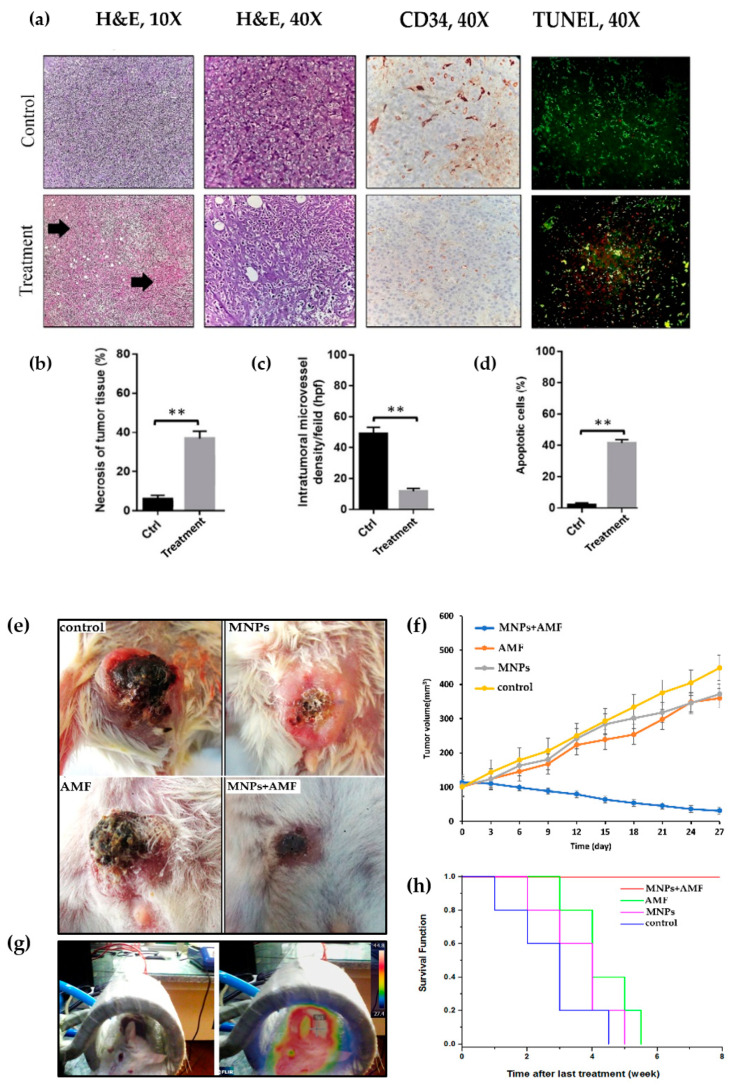
(**a**) Histopathology, immunohistochemistry, and TUNEL assay of breast cancer tissues in control and treated BALB/c mice; (**b**) necrosis of tumor tissue (arrows in histopathological image); (**c**) Immunohistochemical analysis of the cluster of differentiation 34 (CD_34_) marker was performed to determine the angiogenesis in tumor tissues. The brown color represents positive staining of CD_34_ cell marker; (**d**) TUNEL assay was performed to assess the apoptotic cells in the experimental groups. The number of apoptotic-positive cells significantly increased in the treatment group compared to control, red florescent-stained cell nucleus represents apoptotic cells (**p < 0.01); (**e**) tumor shape and appearance in different experimental groups 27 days after the last treatment; (**f**) tumor volume versus time after the last treatment in different groups: magnetic hyperthermia using G_4_@IONPs, only AMF exposure, only G_4_@IONPs injection, and control; (**g**) the tumor temperature was monitored by the forward-looking infrared (FLIR) thermal camera; (**h**) Kaplan-Meier curve showing the survival rate of BALB/c mice over time (8 weeks).

Tumor sections from control and treated mice (MNPs + AMF) were graded histologically using the Nottingham histologic grading system. Many disproportionate tumor cells (anisocytosis), nuclear polymorphism (anisokaryosis, +3), and prominent nucleoli were seen in control animals. Moreover, in the control group, glandular (acinar/tubular) differentiation (GD) was low (+3), and the mitotic index in 10 HPF (400×) was +3. Overall, the tumor in the control group was considered as Grade 3 (poorly differentiated) (Figure 6a). In the treatment group, nuclear polymorphism (+1), GD (+2), and the mitotic index in 10 high power microscopic fields (HPF) (+1) were decreased in comparison to the control group. The tumor in the treatment group was considered as Grade 1 (well-differentiated); furthermore, massive necrosis was seen in breast cancer cells in the treatment group (Figure 6a,b).

### 3.8. Angiogenesis and Apoptosis in Tumor Tissue after Magnetic Hyperthermia Treatment 

The microvessel density (angiogenesis) in treated tumors with magnetic hyperthermia was 13.4 ± 2.4 per HPF compared to that in control tumors (51.3 ± 4.5—*P* < 0.01) (Figure 6a,c). The proportion of apoptotic-positive cells in the treatment group was significantly higher than that in the control group (*P* < 0.01, Figure 6a,d).

### 3.9. Effect of Magnetic Hyperthermia Treatment on Tumor Volume

After the last treatment, tumor volume was measured in all groups every three days for four weeks. The final tumor volume in the group of treatment (30.83 mm^3^) was significantly less than that in the control group (448.11 mm^3^) (Figure 6e,f).

### 3.10. Kaplan–Meier Curve and Survival Rate of BALB/c Mice

Kaplan-Meier curve illustrated the survival rate of BALB/c mice over time in different groups. Five mice in each group were watched for eight weeks after the last treatment. There was no mortality in treated BALB/c mice during this period, while all other mice died during six weeks (Figure 6h). 

## 4. Discussion

Dendrimers are developing polymeric multivalent system with multiple surface functional groups that have been used in many studies for drug delivery and imaging applications [16,47]. Regarding the potentials of dendrimers for theranostic applications, we functionalized IONPs (size of 10 ± 4 nm) with G4 PAMAM dendrimers and mPEG molecules for magnetic hyperthermia treatment of breast cancer. The amine-terminated PAMAM dendrimers attach to the negatively charged membrane of the cells via electrostatic interactions causing cytotoxicity. The results of this study showed that G_4_@IONPs cytotoxicity was negligible even at high concentrations, e.g., 500 μg/mL, that could be due to PEGylation of G_4_@IONPs [48]. PEGylation of MNPs has several advantages, such as additional stability, favorable pharmacokinetics, and enhanced therapeutic activity. Moreover, PEGylation can increase the retention time of nanoparticles [49,50]. The pharmacokinetics, stability, and retention time of G_4_@IONPs were assessed in our previous study [36].

The results of Prussian blue staining and ICP-MS showed that G_4_@IONPs entered into the cytoplasm of the MC_4_L_2_ cells in a concentration-dependent manner. The surface charge of G_4_@IONPs was +35 mV; cationic dendrimers possess a high tendency to interact with the bilayer lipid membrane of cells to consequently increase the cellular uptake [51]. PEGylation of G_4_@IONPs can also alter their cellular uptake via decreasing or preventing the protein adsorption (opsonization) on the G_4_@IONPs surface [52]. Indeed, higher cellular uptake of MNPs can increase the therapeutic effects of magnetic hyperthermia because more MNPs (heat sources) will be close to cell cytoplasmic proteins and nucleus [53]. Magnetic hyperthermia treatment enhanced the cytotoxic effect of G_4_@IONPs and decreased the MC_4_L_2_ cell proliferation; a slight decrease in cell viability in other experimental groups (MNPs and AMF) could be due to G_4_@IONPs incubation and AMF exposure, respectively. 

Apoptosis is a genetically structured cellular death process activated by several internal and external signals. The intrinsic mitochondrial apoptosis pathway is triggered by intracellular stimuli that upregulate the pro-apoptotic Bcl-2 family of proteins, such as Bax, Bad (Bcl-2 associated agonist of cell death), and Bak (Bcl-2 homologous antagonist/killer), leading to the mitochondrial release of cytochrome C [54,55]. This intracellular stress (stimuli) should be strong enough to be able to trigger the apoptosis pathway in the cells; otherwise, some anti-apoptosis molecules would be activated and inhibit the cellular apoptosis procedure. In our study, the magnetic hyperthermia treatment was able to trigger the intrinsic apoptosis pathway and overcome the heat shock proteins (HSP) that cause thermal resistance in cancer cells [56]. TUNEL data approved the MTT viability results; the G_4_@IONPs entered the cancer cells by endocytosis and produced heat after AMF exposure in the group of MNPs + AMF. These small heat sources could then be the powerful intracellular stimuli to trigger the intrinsic apoptosis pathway in the cancer cell (Figure 3h). The results also showed some apoptosis increase (*P* > 0.05) in the group of AMF, which could be due to eddy current induced in the cell dish during the AMF exposure. 

The survivability of treated cancer-bearing BALB/c mice was significantly higher than the untreated ones. This could be because of smaller tumor size in these mice, and, as a result, they could move better in the cage and access to more food and water. Besides that, general health was better in treated mice that helped them to survive longer. The body weight did not show any significant variation during the experimental period that showed our MNPs did not alter the body metabolism in mice. In a similar study, Malik et al. also reported that injecting PAMAM dendrimer (95 mg/kg) into B16F10 tumor-bearing mice (three times per day) did not alter their weight [57].

Our results showed that the tumor volume in treated mice decreased significantly to 23.7% of the initial volume (*V_t0_*) over 27 days. This ratio (*V/V_t0_*) was 448% in control animals. Furthermore, tumor volume reduction in other mice (groups of MNPs and AMF) compared to control could be due to intratumoral G_4_@IONPs injection or AMF exposure. Tumor volume is one of the most important parameters to demonstrate the efficacy of magnetic hyperthermia treatment; therefore, many studies reported the final tumor volume, as well as histopathological outcomes. For instance, Alexanian et al. injected superparamagnetic IONP (SPION) linked to PEG and folic acid (FA-PEG-SPION) into mice intravenously and placed them in an alternating current (AC) magnetic field (8 kA/m and 230 kHz) for 20 min. They reported that tumor volume in treated mice was one-tenth of control ones 35 days after the last treatment [52]. 

Tumor regression after magnetic hyperthermia treatment could be due to increasing the cancer cell apoptosis as both in vitro and H&E results showed that the number of apoptotic cells in the group of MNPs + AMF was significantly more than that in the control group. Furthermore, magnetic hyperthermia treatment decreased the intratumoral microvessel density, which could be another reason for tumor shrinkage in treated mice. 

Regarding the effect of magnetic hyperthermia treatment on tumor vascularization, Kossatz et al. investigated the degree of angiogenesis in tumor tissues using the CD_31_ indicator [23]. To this end, IONPs (25 mg Fe/100 mm^3^) were injected intratumorally, and, after 24 h, nude mice were exposed to an AMF (15.4 kA/m and 435 kHz). Their results showed that the rate of angiogenesis in treated tumors decreased 28 days after the first treatment. We also obtained similar results regarding tumor angiogenesis suppression with a lower amount of iron injected into the tumor (5 mg/mL). In our previous study, we showed that G_4_@IONPs were highly capable of converting AMF energy to heat [21]. Therefore, to generate a sufficient amount of heat inside the tumor, a lesser amount of MNPs was used in our study. 

The highest temperature (max 45 ℃), and also temperature uniformity, in the tumor were monitored by a FLIR thermal camera system (Figure 6g) and based on this data, the location and number of MNPs injection sites were determined. The uniformity of heat distribution in the tumor is a determining factor in magnetic hyperthermia treatment [58]. Hence, multiple injection sites (four sites) were applied to obtain a more homogenous MNPs distribution in the tumor. G_4_@IONPs (0.1 mL) were gently injected intratumoral using an insulin syringe (Ultra-Fine needle) and after 15 min, the animal was transferred to the AMF coil. No leakage of the MNPs solutions back out was seen after the syringe needle was removed from the tumor. 

Our results revealed that treated breast cancer cells (MNPs + AMF) could not progress to invasive ones over time, which could be due to higher apoptosis and necrosis in these cells. Histopathological evaluations were performed by a single-blinded pathologist and Nottingham Histologic Scoring System was used to assess the breast tumor grade. The tumors in treated and control mice were in grades 1 and 3, respectively. In grade 1, the cancer cells look similar to normal cells and usually are well differentiated. Grade 3 breast cancer is invasive, tumors may be larger, and cancer has spread to the lymph nodes [59,60]. The treatment could induce differentiation in breast cancer cells, in addition to inhibiting tumor growth. Histopathological studies (H&E staining) also revealed a higher amount of necrotic cells at the center of treated tumors. Consequently, stem cells at the center of the tumor that are mainly responsible for metastasis and tumor progression would be necrotic after magnetic hyperthermia treatment. This can explain the lack of metastasis and invasive cancer cells in lung and liver tissues in treated animals. On the other hand, it has been indicated that extracellular HSPs trigger antitumor immunity during tumor cell necrosis. Such induced immunity also promotes tumor regression [61].

Overall, magnetic hyperthermia treatment using G_4_@IONPs increased the cellular apoptosis via the intrinsic apoptosis pathway and at the same time cut the tumor blood supply by decreasing the tumor angiogenesis. These factors inhibited the tumor growth and progression; consequently, cancer cells did not spread and develop metastasis in healthy tissues. The present study has some limitations regarding the MNPs distribution and quantity in the tumor after injection; first, the precise distribution of G_4_@IONPs could have investigated by magnetic resonance imaging (MRI), confocal microscopy, and Prussian blue staining; second, the iron content in the tumor was not measured after intratumoral G_4_@IONPs injection.

## 5. Conclusions

The nanocomposites presented in this study were well-suited for magnetic hyperthermia treatment of breast cancer. The toxicity assay revealed the biocompatibility of G_4_@IONPs even at high concentrations up to 500 µg/mL. Breast cancer cell viability decreased significantly after magnetic hyperthermia treatment. Furthermore, cellular apoptosis increased in treated cancer cells; real time-PCR results also showed that magnetic hyperthermia treatment was able to regulate the expression of the apoptosis-related genes. The dendrimer functionalized IONPs presented here also showed promising outcomes for breast cancer treatment in BALB/c mice. The magnetic hyperthermia treatment decreased tumor mammary gland growth via suppressing the tumor angiogenesis and increasing the cellular necrosis. All in all, G_4_@IONPs seem to be suitable nanostructures for use in magnetic hyperthermia cancer treatment due to their biocompatibility, stability, and unique structure for the conjugation of biomolecules. For future studies, anti-cancer drugs and imaging contrast agents can be conjugated to the dendrimer coating for cancer theranostics applications.

## Figures and Tables

**Table 1:** Different types of nanoparticles used in the magnetic hyperthermia treatment for cancer-bearing mice.

Study	MNPs Core	MNPs Size	Coating	Treatment Time	MNPs Concentration/Type of Injection	Tumor Model	AMF	Results
Hayashi et al. [22]	Fe_3_O_4_	10.5 nm	PPy-PEG-FA Dox	20 min	5 mg/kg; intratumoral injection	Multiple myeloma	8 kA/m 230 kHz	The combination of magnetic hyperthermia treatment and chemotherapy completely cured the tumor without any recurrence.
Kossatz et al. [23]	SPIONs	12 ± 3 nm	N6L or/and DOX	60 min	0.25 mg Fe/100 mm^3^; intratumoral injection	Breast	15.4 kA/m 435 kHz	Substantial tumor growth inhibition up to 40% and complete tumor regression were seen after magnetic hyperthermia treatment.
Haghniaz et al. [24]	La_0.7_sr_0.3_MnO_3_	25–50 nm	dextran	20 min	5 mg/100 μL saline; intratumoral injection	Melanoma	700 A 8000 W 365 kHz	Treatment inhibited tumor growth (84%) and increased animal survival (50%). In addition, levels of caspase-3 and caspase-6 also increased after treatment.
Lee et al. [25]	CoFe_2_O_4_	15 nm	MnFe_2_O_4_	10 min	75 mg; intratumoral injection	Glioblastoma	37.3 kA/m 500 kHz	The tumor was clearly eliminated in 18 days after treatment.
Li et al. [26]	Fe_3_O_4_	22 nm	anti-HER2, 5-FU and PEG	15 min	500 mg/mL iron; systematic injection	Bladder carcinoma	33 kA/m 1.3 MHz	Prominent tumor remission was seen after hyperthermia and chemotherapy.
Rabias et al. [27]	Fe_2_O_3_	10–12 nm	dextran	20 min	150 μL; intratumoral injection	Glioma	11 kA/m 150 kHz	Significant tumor tissue damage and dissolution were seen after treatment.
Bae et al. [28]	Fe_3_O_4_	30 nm	Chitosan-DOPA	20 min	375 μg Fe/kg; Intratumoral injection	Lung carcinoma	660 A/m 1 MHz	The tumor volume decreased substantially by about 70%.
Ling et al. [29]	Fe_3_O_4_	20–50 nm	PMMA	3 min	0.1 mL; intratumoral injection	Breast	28.6 A 626 kHz	Tumor volume decreased within 15 days after treatment.
Arriortua et al. [30]	Fe_3_O_4_	19 ± 2 nm	RGD peptide	>21 min	1–1.5 mg Fe/mL; systemic injection	Colon adenocarcinoma	14 kA/m 606 kHz	Tumor necrosis was observed. Approximately whole tumor tissue was demolished in some animals, others showed very low damage in tumor tissue.
Ohtake et al. [31]	Fe(Salen)	200 nm	Salen	60 min	0.12–0.60 mg/body; intratumoral injection	Glioblastoma	335. 4 A 280 kHz	The tumor size was decreased, by 80–90%, in treatment group after 4 weeks.
Sato et al. [32]	Fe(Salen)	200 nm	-	30 min	50 mM; intratumoral injection	Tongue	250 A 308 kHz	The tumor volume significantly decreased (223 ± 80.6%). The tumor almost completely disappeared after one week.
Yang et al. [33]	Fe_3_O_4_	-	PLGA	3 min	100 μL; intratumoral injection	Hepatic carcinoma	28.6 A 626 kHz	Coagulative necrosis was seen in cancer tissues after treatment. In addition, anti-tumor immune system was activated in treated mice and promoted apoptosis in tumor cells.
Zhang et al. [34]	Fe_3_O_4_	18 nm	PPZ polymer	60 min	0.8 µL/mm^3^; intratumoral injection	Glioblastoma	13.3 kA/m 366 kHz	The tumor size was significantly smaller than the control 25 days after the last treatment. Pyknosis, karyorrhexis, and apoptosis were seen in treated tumor tissues.
Hayashi et al. [35]	SPIONs	7−9 nm	PEG and FA	20 min	48 μmol Fe/kg; systematic injection	Multiple myeloma	8 kA/m 230 kHz	Tumor volume in treated mice was one-tenth of control in 35 days after treatment

Abbreviations: SPIONs, superparamagnetic iron oxide nanoparticle; DOX, doxorubicin; HER2, human epidermal growth factor receptor; FU, fluorouracil; PPy, polypyrrole; FA, folic acid; DOPA, dihydroxyphenylalanine; PMMA, polymethyl methacrylate; RGD, arginylglycylaspartic acid; PLGA, poly(lactic-co-glycolic acid); PPZ, polyorganophosphazene.

**Table 2:** Oligonucleotide sequences of interest and reference genes.

Gene	Sequences (5′ → 3′)	Product Size, bp
GAPDH-F	AAGTTCAACGGCACAGTCAAGG	22
GAPDH-R	CATACTCAGCACCAGCATCACC	22
Bax-F	AGGGTGGCTGGGAAGGC	17
Bax-R	TGAGCGAGGCGGTGAGG	17
Bcl2-F	ATCGCTCTGTGGATGACTGAGTAC	24
Bcl2-R	AGAGACAGCCAGGAGAAATCAAAC	24
